# Dysregulation of AKT3 along with a small panel of mRNAs stratifies high-grade serous ovarian cancer from both normal epithelia and benign tumor tissues

**DOI:** 10.18632/genesandcancer.164

**Published:** 2017-11

**Authors:** Pourya Naderi Yeganeh, Christine Richardson, Zahra Bahrani-Mostafavi, David L. Tait, M. Taghi Mostafavi

**Affiliations:** ^1^ College of Computing and Informatics, University of North Carolina at Charlotte, Charlotte, NC, USA; ^2^ Department of Biological Sciences, University of North Carolina at Charlotte, Charlotte, NC, USA; ^3^ College of Health and Human Services, University of North Carolina at Charlotte, Charlotte, NC, USA; ^4^ Division of Gynecological Oncology and Obstetrics, Levine Cancer Institute, Carolinas Medical Center, Charlotte, NC, USA

**Keywords:** ovarian cancer, AKT3, genomic analysis, high-grade serous ovarian cancer, biomarker discovery

## Abstract

Screening methods of High-Grade Serous Ovarian Cancer (HGSOC) lack specificity and sensitivity, partly due to benign tumors producing false-positive findings. We utilized a differential expression analysis pipeline on malignant tumor (MT) and normal epithelial (NE) samples, and also filtered the results to discriminate between MT and benign tumor (BT). We report that a panel of 26 dysregulated genes stratifies MT from both BT and NE. We further validated our findings by utilizing unsupervised clustering methods on two independent datasets. We show that the 26-genes panel completely distinguishes HGSOC from NE, and produces a more accurate classification between HGSOC and BT. Pathway analysis reveals that AKT3 is of particular significance, because of its high fold change and appearance in the majority of the dysregulated pathways. mRNA patterns of AKT3 suggest essential connections with tumor growth and metastasis, as well as a strong biomarker potential when used with 3 other genes (PTTG1, MND1, CENPF). Our results show that dysregulation of the 26-mRNA signature panel provides an evidence of malignancy and contribute to the design of a high specificity biomarker panel for detection of HGSOC, potentially in an early more curable stage.

## INTRODUCTION

Ovarian cancer is the leading cause of death among gynecological cancers and early diagnosis is one of the key challenges in reducing the mortality rate [1-3]. Early detection strategies of ovarian cancer lackrequired specificity and sensitivity, leading to diagnosis inadvanced and more lethal stages in 75% of patients [4, 5]. For example, elevations of the biomarker Cancer Antigen125 (CA125) produce a relatively small true positive ratefor early stage ovarian cancers, while the false positiverates are high for patients with benign tumors and non-cancerous subjects [5, 6]. Major clinical trials -- Prostate, Lung, Colon, and Ovarian (PLCO) screening trials, and UK Collaborative Trial of Ovarian Cancer Screening (UKTOCS) – indicate that the current screening strategies do not contribute to the reduction of mortality rates [7, 8]. Not surprisingly, the design and discovery of credible biomarker panels for detection of ovarian cancer has emerged as a formidable task.

To date, genome-scale comparative studies have considerably contributed to the discovery of new markers, therapeutic targets, cancer subtypes, and origins of ovarian cancer. [9-13]. The utilization of genomic scale analyses has also substantially benefited the characterization of malignant tumors [14, 15]. However, many of the genetic and pathological features associated with cancer also occur in benign tumors, including some of the hallmarks of cancer, such as evading growth suppressors and resisting cell death [14-16]. The molecular and pathological resemblance between malignant and non-malignant tumors is a barrier in the design of cancer-focused detection and treatment approaches [16]. Given these challenges, the identification of malignancy-specific molecular signatures plays a critical role in the disease diagnosis and management.

This study focuses on identification of a molecular signature panel in High-Grade Serous Ovarian Cancer (HGSOC) which constitutes up to 80% of the cases of ovarian cancer [17]. We focus on the discovery of a small signature panel of mRNA markers that can differentiate between malignant and benign tumors. We hypothesize that comparative mRNA analysis of ovarian tissues would reveal critical signatures of malignancy and tumor progression in HGSOC. To probe our hypothesis, we investigated the gene expression profiles of distinct ovarian tissues by utilizing Laser Capture Microdissection (LCM) for high sample purity [18]. We analyzed the mRNA expression levels of normal ovarian epithelia (NE), benign ovarian tumors (BT), and HGSOC malignant tumors (MT). Analyses were performed to capture the differential expressions patterns, similar to Bowen et al. [9] who utilized a related design and identified ovarian surface epithelial markers in the cancer tumors. Our experimental design uniquely included BT samples which allowed for a more comprehensive molecular characterization of the different pathological states [16]. Our results identify a small signature mRNA panel of 26 dysregulated genes that stratifies MT from both BT and NE. Validity of the identified panel was verified by using classification algorithms on two publicly available independent datasets (GSE9899 and GSE14407) [9, 12]. To determine the functional properties of the dysregulated genes in the active programs of HGSOC, we compared results from pathway enrichments and the mRNA dysregulation between MT and BT.

## RESULTS

### Differential mRNA expression analysis of normal epithelia versus malignant tumors

Analysis of MT and NE samples (Table 1) detects 216 significantly differentially expressed genes, 100 of which are upregulated and 116 are downregulated (supplementary table). Table 2 details the 20 upregulated genes with greatest fold changes as well as the 20 downregulated genes with greatest fold changes. The analysis detects dysregulation of genes previously associated with HGSOC – such as Paired box 8 (PAX8), Paternally expressed 3 (PEG3), Survivin (BIRC5), Meis homeobox 1 (MEIS1), and Homeobox C6 (HOXC6) - as well as unreported ones, such as Meiotic nuclear divisions 1 (MND1), and Protein Kinase B, Gamma (AKT3), (Table 2 and supplementary table) [19-21].

**Table 1:** Sample information used in the study

Sample ID**	Tissue Type	Primary Pathology	Stage*
I	Benign tumor tissue	Serous Cyst	---
II	Benign tumor tissue	Serous Cystadenofibroma	---
III	Benign tumor tissue	Serous Cyst	---
IV	Benign tumor tissue	Serous Cystadenofibroma	---
V	Benign tumor tissue	Serous Cyst	---
VI	Benign tumor tissue	Serous Cyst	---
VII	Malignant tumor tissue	Serous Carcinoma	II
VIII	Malignant tumor tissue	Serous Carcinoma	IIIc
IX	Malignant tumor tissue	Serous Carcinoma	IIIc
X	Malignant tumor tissue	Serous Carcinoma	IIIc
XI	Malignant tumor tissue	Serous Carcinoma	NA^
XII	Malignant tumor tissue	Serous Carcinoma	IIIc
XIII	Malignant tumor tissue	Serous Carcinoma	IIIc
XIV	Malignant tumor tissue	Serous Carcinoma	IIIc
XV	Normal ovarian epithelia	Non-tumorous	---
XVI	Normal ovarian epithelia	Malignant Serous Carcinoma	II
XVII	Normal ovarian epithelia	Serous Cystadenofibroma	---
XVIII	Normal ovarian epithelia	Simple Serous Cyst	---
XIX	Normal ovarian epithelia	Metastatic Serous Carcinoma	IIIc
XX	Normal ovarian epithelia	Metastatic Serous Carcinoma	IIIc
XXI	Normal ovarian epithelia	Serous Cystadenofibroma	---
XXII	Normal ovarian epithelia	Metastatic Serous Carcinoma	---
XXIII	Normal ovarian epithelia	Metastatic Serous Carcinoma	---
XXIV	Normal ovarian epithelia	Metastatic Serous Carcinoma	IIIc

* Indicates the stage of the patients at the time of sample collection according to The International Federation of Gynecology and Obstetrics.

^ NA indicates unavailability of information regarding sample.

** Data from this study is accessible through NCBI's Gene Expression Omnibus through dataset GSE 29156.

**Table 2:** Top 20 downregulated and upregulated genes in MT compared to NE

Downregulated Genes				Upregulated Genes			
Gene Symbol - Description	Ref Seq	P-value^	FC^^	Gene Symbol - Description	Ref Seq	P-value^	FC^^
*DCN – decorin	NM_133506	2.29E-05	-13.8	PTTG1 - pituitary tumor-transforming 1	ENST00000352433	1.96E-06	28.8
C7 - complement component 7	NM_000587	2.78E-04	-10.0	MAL2 - mal, T-cell differentiation protein 2	NM_052886	4.12E-08	26.5
EFEMP1 - EGF containing fibulin-like extracellular	NM_001039348	9.18E-07	-9.09	CP - ceruloplasmin	NM_000096	6.64E-06	17.4
*THBS1-thrombospondin 1	ENST00000260356	9.84E-05	-8.93	ESRP1 - epithelial splicing regulatory protein 1	NM_017697	3.47E-06	14.6
PEG3 - paternally expressed 3	NM_006210	4.34E-04	-8.11	LPAR3 - lysophosphatidic acid receptor 3	NM_012152	6.50E-07	12.2
MGP - matrix Gla protein	NM_001190839	3.94E-05	-5.68	CD24 - CD24 molecule	NM_013230	2.27E-06	10.3
CDH11- cadherin 11	ENST00000268603	2.24E-04	-5.67	VAMP8 – vesicle associated membrane protein 8	NM_003761	3.41E-05	9.8
*AKT3 - protein kinase B, gamma	NM_005465	2.99E-04	-4.68	MECOM - MDS1 and EVI1 complex locus	NM_001105077	2.61E-06	8.69
ANTXR1 – anthrax toxin receptor	NM_032208	7.10E-06	-4.64	RPL39L - ribosomal protein L39-like	ENST00000296277	0.000168	7.11
OLFML1 - olfactomedin-like 1	ENST00000530135	4.79E-06	-4.39	WFDC2 - WAP four-disulfide core domain 2	ENST00000342873	4.99E-06	7.11
ANTXR2 – anthrax toxin receptor 2	ENST00000403729	1.48E-05	-4.09	SLC38A1 - solute carrier family 38, member 1	NM_030674	2.13E-05	6.41
CALD1- caldesmon 1	NM_033138	7.66E-06	-4.08	SPINT2 - serine peptidase inhibitor, Kunitz type, 2	NM_021102	3.68E-06	6.36
NEXN - nexilin (F actin binding protein)	NM_144573	3.55E-04	-4.07	CLDN4 - claudin 4	ENST00000435050	8.17E-09	6.13
PTGIS - prostaglandin I2 (prostacyclin) synthase	NM_000961	6.21E-04	-3.88	CENPF- centromere protein F	NM_016343	0.000209	5.90
PLS3 - plastin 3	NM_005032	6.44E-04	-3.85	DPY30 - dpy-30 homolog	NM_032574	0.000260	5.79
RHOBTB3 - Rho-related BTB domain containing 3	NM_014899	4.78E-04	-3.76	XPR1 - xenotropic and polytropic retrovirus	NM_004736	1.61E-05	5.76
SDC2 - syndecan 2	NM_002998	1.23E-04	-3.68	EPCAM - epithelial cell adhesion molecule	NM_002354	3.34E-05	5.65
CCDC80 - coiled-coil domain containing 80	ENST00000206423	1.16E-04	-3.61	BIRC5 - Survivin	AF077350	0.000429	5.61
SULF2 - sulfatase 2	ENST00000359930	1.42E-04	-3.61	SCNN1A - sodium channel, non-voltage-gated 1	NM_001038	7.50E-05	5.48
TRPC1 - transient receptor potential cation channel	NM_001251845	3.53E-05	-3.60	DSP - desmoplakin	ENST00000379802	0.000238	5.41
COL14A1 - ollagen, type XIV, alpha 1	NM_021110	3.11E-05	-3.55	MEIS1 - Meis homeobox 1	NM_002398	5.20E-05	5.20

^ P-values are calculated based on ANOVA method and all of the p-value were subjected to multiple hypothesis testing criteria (FDR<0.05).

^^ Fold Change of Malignant versus Normal

* Genes are also present in the dysregulation network displayed in Figure 6.

### Dysregulated mRNA expressions between benign and malignant tumors

**Figure 1:**
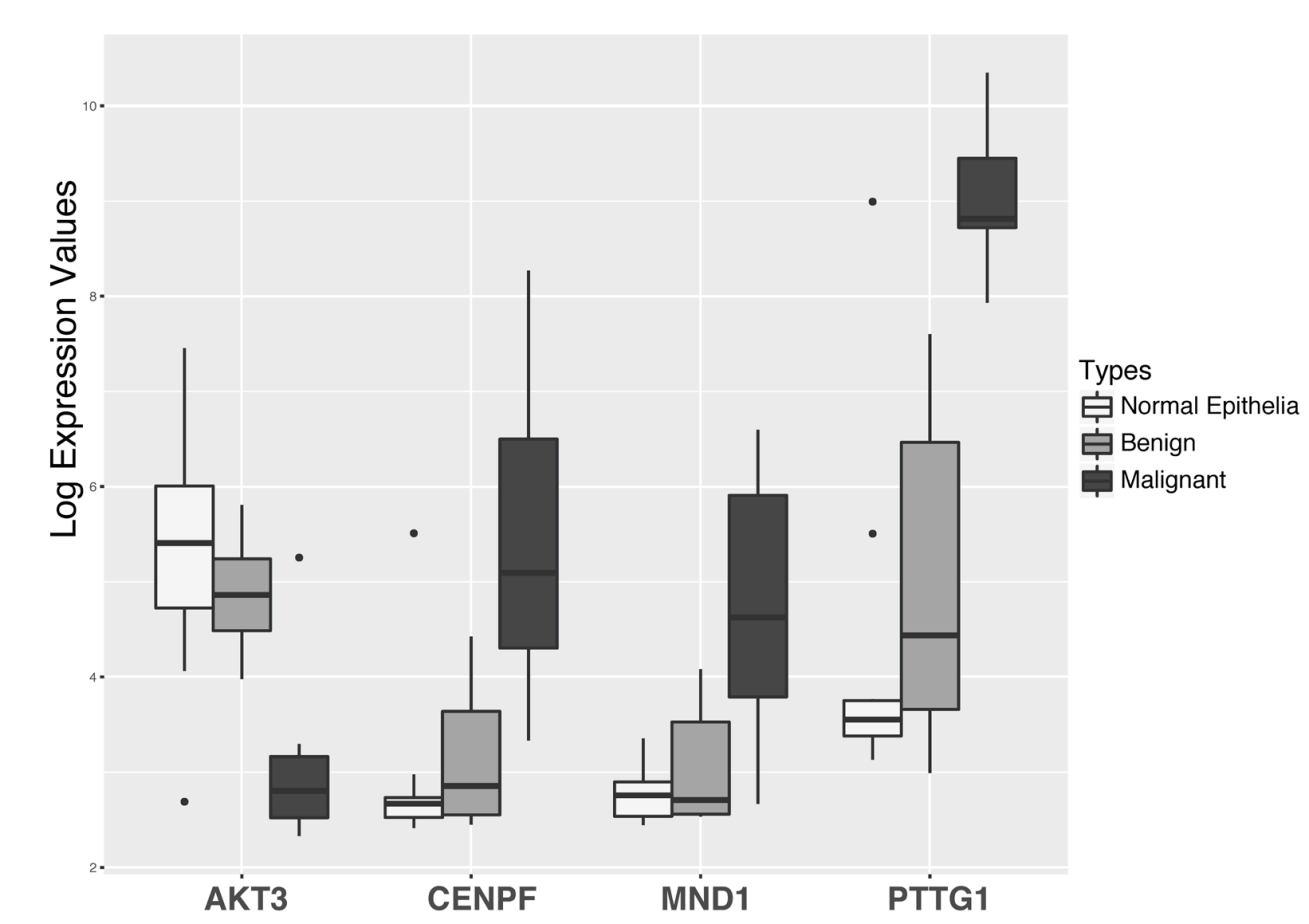
The expression patterns of AKT3, CENPF, MND1, and PTTG1 distinguish between normal ovarian epithelia, benign tumors, and malignant tumors The expression data are obtained using microarray signals from LCM samples. The graph displays the differentially expressed genes with fold change magnitude more than 3. Further confirmation is displayed in Figure 2.

At the initial step of the analysis pipeline, 216 dysregulated genes were found between MT and NE. The dysregulated genes were then tested in another specific comparison pipeline. Of the 216 dysregulated genes 26 were detected as differentially expressed between MT and BT (Table 3). Among the 26 genes, AKT3, Human Securin encoding gene (PTTG1), Centromere protein F (CENPF), and MND1 exhibit fold change magnitudes of greater than 3 (Figure 1, Table 3). In MT vs. NE setting, AKT3 is downregulated by 4.68 folds (p-value < 2.99 E-04), and PTTG1, CENPF, and MND1 are all upregulated by 28.8 fold (p-value < 1.96 E-06), 5.9 fold (p-value < 2.1 E-04), and 3.9 fold (p-value < 1.29 E-04), respectively. In MT vs. BT setting, the mRNA dysregulation of these four genes (AKT3, PTTG1, CENPF, and MND1) yield a similar statistically significant pattern (Table 3).

**Table 3:** List of the genes differentially expressed between MT compared to both BT and NE

Upregulated Genes				Downregulated Genes			
Gene Symbol - Description	Ref Seq	P-value^	FC^^	Gene Symbol - Description	Ref Seq	P-value^	FC^^
CDC7 - cell division cycle 7 homolog	NM_001134420	6.58E-05	2.64	BNIP3L- BCL2/ adenovirus E1B 19kDa interacting protein 3-like	ENST00000380629	0.00250	-2.18
**PTTG1 - pituitary tumor-transforming 1**	**ENST00000352433**	**9.69E-05**	**16.9**	DKK3 - dickkopf 3 homolog	AF400439	0.00320	-1.52
FDPS - farnesyl diphosphate synthase	NM_001135821	9.90E-05	2.51	*AKT3 – Protein Kinase B, gamma	**NM_005465**	**0.00565**	**-3.50**
*RAB11FIP4 - RAB11 family interacting protein 4	NM_032932	0.000201	1.42				
PITRM1 - pitrilysin metallopeptidase 1	NM_001242307	0.000391	2.36				
NAA40 - N(alpha)-acetyltransferase 40	ENST00000377793	0.000585	1.74				
*BCL11A - B-cell CLL/lymphoma 11A	NM_022893	0.000656	1.72				
PUS7 - pseudouridylate synthase 7 homolog	ENST00000356362	0.000889	1.84				
BIRC5 - Survivin	AF077350	0.000925	1.93				
TFB2M - transcription factor B2, mitochondrial	ENST00000366514	0.00110	2.23				
PCGF1 - polycomb group ring finger 1	ENST00000233630	0.00136	2.05				
EZH2 - Enhancer of zeste homolog 2	NM_001203247	0.00173	2.53				
**CENPF – Centromere protein F**	**NM_016343**	**0.00181**	**5.01**				
RACGAP1- Rac GTPase activating protein 1	NM_013277	0.00185	2.69				
**MND1 - Meiotic nuclear divisions 1**	**NR_045605**	**0.0020**	**3.20**				
UBAP2L - ubiquitin associated protein 2-like	NM_014847	0.0021	2.58				
HJURP - Holliday junction recognition protein	NM_018410	0.00307	2.01				
LRRTM1 - LRRN4 C-terminal like	ENST00000433224	0.00385	1.70				
MRPS18A-mitochondrial ribosomal protein S18A	NM_018135	0.00394	2.29				
PRKDC - protein kinase, DNA-activated, catalytic polypeptide	NM_006904	0.00447	2.24				
POGK - pogo transposable element with KRAB domain	ENST00000367875	0.00479	1.74				
TMEM206 -transmembrane protein 206	ENST00000261455	0.00527	1.40				
CDK16 - cyclin-dependent kinase 16	NM_006201	0.00556	2.26				

* Genes are also present in the dysregulation network displayed in Figure 6.

Bold indicates fold change more than 3; FC^^ indicates fold change of Malignant versus Benign

**Figure 2:**
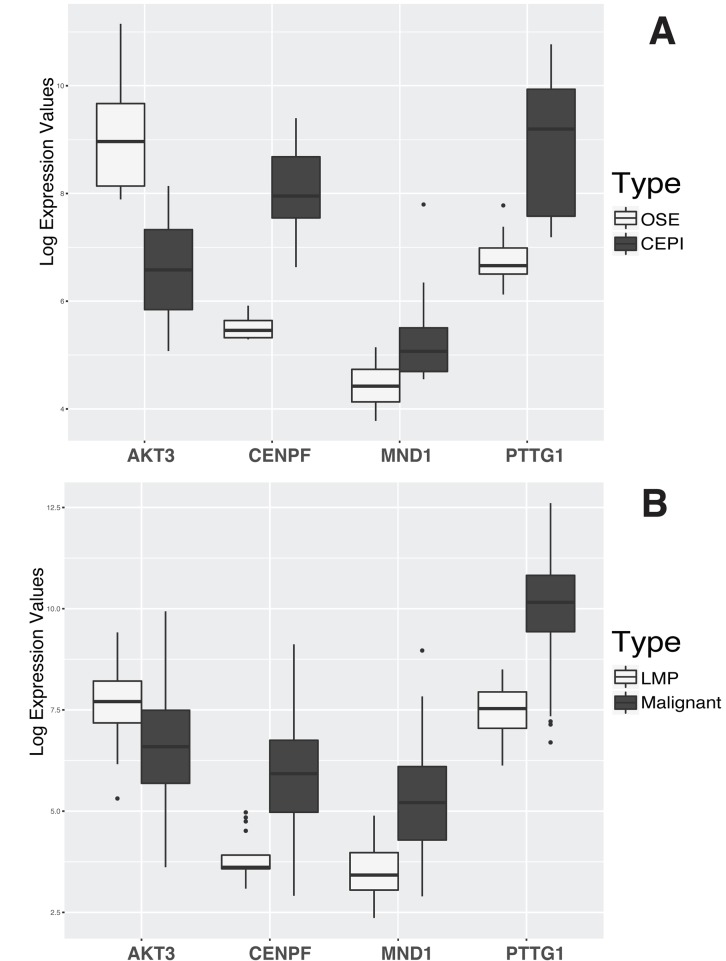
Evaluation of AKT3, CENPF, PTTG1, and MND1 differential expression using two independent datasets A) Box plots are based on Tothill's data using 264 microarray samples of ovarian tumors with low potential malignant (LMP) samples andcancer tumors [12]. The lighter-shade boxplots represent the mRNA expression levels in LMP. Darker-shade boxplots represent the mRNA expression levels in malignant tumors. B) Box plots are based on Bowen's data with 12 LCM ovarian epithelia samples and 12 cancer tissues [9]. The lighter-shade boxplots represent the mRNA expression levels in normal ovarian epithelial tissues and the darker-shade boxplots represent mRNA expression levels in cancer tumors. Additional information on microarray analysis on both datasets can be found in supplementary material

Cross-examination of expression of the set of four genes provides additional evidence for their dysregulation in HGSOC. Two independent and publicly available datasets by Tothill et al. (GSE9899) and Bowen et al. (GSE14407) (Figure 2) [9, 12] were utilized. In malignant tumors compared to Low Malignant Potential (LMP) tumors in GSE9899 dataset, AKT3 is downregulated by 2.1 folds (p-value < 0.0012); PTTG1, MND1, and CENPF are upregulated with 5.9 (p-value < 2.83 E-19), 4.12 (p-value 9.9 E-11), and 3.17 folds (p-value < 1.67E- 08), respectively. The dysregulation patterns persist in LCM-collected normal epithelia and malignant epithelial ovarian tissues (from GSE14407). AKT3 is downregulated by 5.7 folds (p-value < 4.11 E-06); PTTG1, MND1, and CENPF are upregulated with 4.2 (p-value < 3.52 E-05), 1.86 (p-value < 0.008), and 5.78 folds (p-value < 1.87 E-09), respectively. Although the experimental design and samples are different in each study, the displayed results validate the findings. The test datasets contain control samples from normal and benign ovarian tissues, and provide a suitable sample composition for validating our findings.

Enrichment analysis results of the 26 differentially expressed genes between MT and BT detects the terms *cell cycle, regulation of mitotic cell cycle phase transition, regulation of cell cycle phase transition, E2F cell cycle related transcription factors, and G2/M checkpoint inhallmarks.* Common biological knowledge assumes that the malignant cells exhibit attributes indicating invasion and rapid cell growth compared to benign cells. PTTG1, CENPF, BIRC5, Enhancer of zeste homolog 2 (EZH2), Rac GTPase activating protein 1 (RACGAP1), and cell division cycle 7 homolog (CDC7) are associated with G2M checkpoint (p-value < 3.16 E-6). Similarly, BIRC5, EZH2, RACGAP1, PTTG1, and protein kinase, DNA-activated, catalytic polypeptide (PRKDC) are associated with E2F transcription factor cell cycle-related targets (p-value < 1.06 E-4).

### A panel of 26 dysregulated genes distinguishes HGSOC tumors from normal and benign ovarian samples

**Figure 3:**
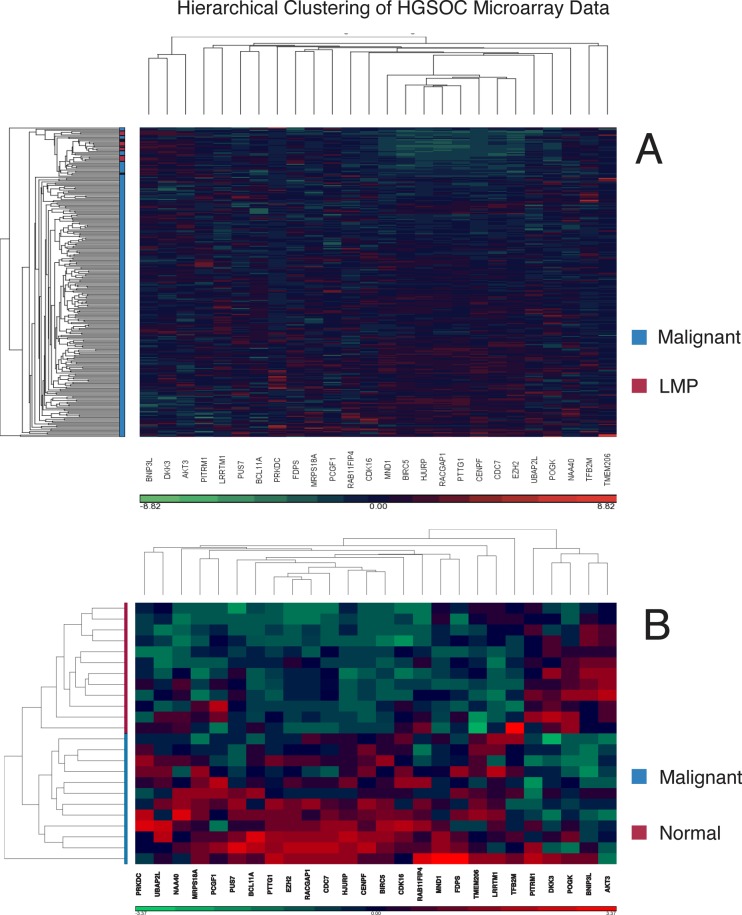
Hierarchical clustering of the 26-genes signature panel and the independent datasets A) When applied to GSE9899 dataset by Tothill et al., LMP samples are clustered closely. B) Hierarchical clustering clearly distinguishes between normal epithelia and cancer tissues. Further assessment of the gene panel using k-means algorithm is displayed in Figures 4 and 5.

The two datasets (GSE14407 and GSE9899) were used to investigate the utility of the 26 dysregulated genes (Table 3) as a mRNA panel to stratify malignant samples from benign and normal samples. Unsupervised hierarchical clustering of the mRNA panel on GSE9899 shows that LMP samples are clustered close together and separated from malignant samples (Figure 3A). Using a similar procedure on GSE14407 shows a clear distinction between the normal epithelial samples and malignant samples (Figure 3B).

**Figure 4:**
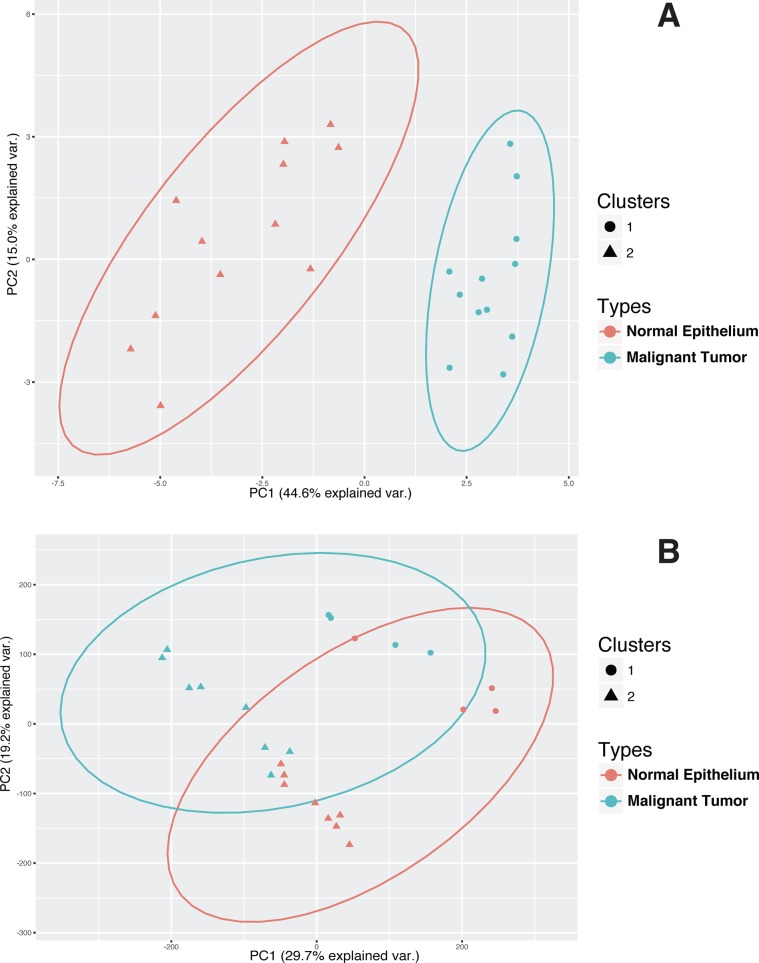
Unsupervised classification of HGSOC and normal epithelial samples Use of the 26-gene dysregulation panel (Table 3) improves the unsupervised classification of an independent sample set from GSE14407 data. The plot shows the projection of the samples into the first two principal components. A) Two clusters distinctly represent the two tissue types and the groups are completely separated when only the 26-gene dysregulation panel is used. Cluster 1 represents 100% normal samples and cluster 2 represents 100% malignant samples. The use of all genes for PCA and k-means cannot stratify between the samples, and the clusters represent mixed tissue types. In particular, each cluster is composed of 50% malignant and 50% normal samples (Table 4). Overlap of the confidence ellipses indicates that using PCA is not sufficient to distinguish between the two tissue types. The data-point shapes indicate the cluster memberships designated using k-means algorithm for the unsupervised clustering. The ellipses represent the 2-dimentional 95% confidence interval for each tissue type.

Principal Component Analysis (PCA), followed by k-means clustering, was used to further assess the utility of the mRNA panel. PCA of GSE14407 samples shows that the use of the 26-genes mRNA panel is sufficient to stratify normal epithelial tissues from HGSOC (Table 4). The unsupervised k-means clustering separates the tissue types into two distinct groups with no overlap between their 95% confidence ellipses, when only using the two first principal components (Figure 4). In contrast, a similar procedure fails to distinguish between the tissue types when the mRNA signature panel is not used for PCA; and finds an overlap between the 95% confidence ellipses. When using the mRNA panel, the first two principal components explain more than 59.6% of the variance in the data, up from 48.9% when using all the genes for PCA.

**Table 4:** Unsupervised k-means clustering of the two independent datasets, GSE9899 and GSE14407, using a 2-dimensional PCA projection of the dysregulated 26-gene panel

		PCA Dysregulated Genes		PCA All Genes	
		Cluster 1	Cluster 2	Cluster 1	Cluster 2
Bowen et al. GSE14407	Normal	12	0	4	8
	Cancer	0	12	4	8
Tothill et al. GSE9899	LMP	0	18	8	10
	Cancer	179	67	93	153

**Figure 5:**
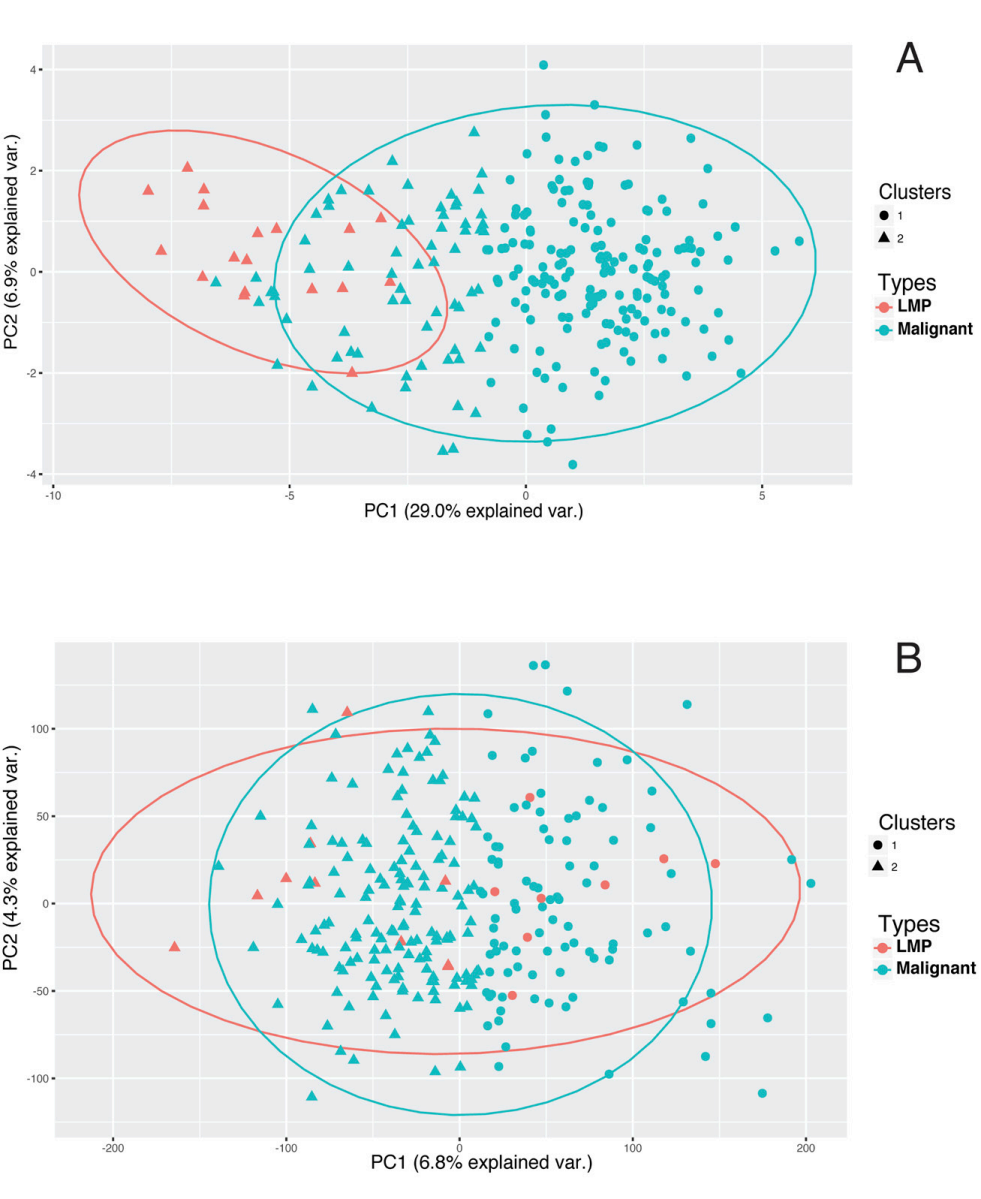
Unsupervised classification of HGSOC and LMPsamples The use of the 26-gene dysregulation panel (Table 3) improves the classification of an independent sample set from GSE9899 data. The samples are projected into the first two principal components. A) By using the 26-gene signature panel, unsupervised clustering stratifies LMP samples into the same group while the other group consists only of malignant samples (Table 4). In particular, LMP samples are allocated only to the cluster 2. Also, the clusters 1 and 2 represent approximately 72% and 28% of the malignant samples, respectively. In this case, the two principal components explain 35.9% of the variance in the data B) The use of all genes for PCA and k-means cannot stratify between the samples, and the clusters represents mixed tissue types as the confidence ellipsoids majorly overlap (Table 4). In particular, LMP samples are allocated to both clusters. Also, the clusters 1 and 2 represent approximately 38% and 62% of the malignant samples, respectively. In this case, the amount of data variance explained by the first two principal components drops to 11.1%. Data-point shapes indicate the cluster memberships designated using k-means algorithm. The ellipses represent the 2-dimentional 95% confidence interval for each tissue type.

PCA followed by k-means clustering on GSE9899 dataset shows that using the dysregulated mRNA panel produces a stronger classification compared to when using all genes (Figure 5). k-means clustering classifies LMP samples into the same group and associates a larger fraction of the malignant tumors to another group when using the 26-genes mRNA panel as a base. In contrast, using all genes' transcripts fails to discriminate between the two groups and distributes the tissue types in both of the clusters (Table 4). Also, using the mRNA panel increases the variance in data explained by PCA. The first two principal components explain 35.9% of the variance when using the 26-panel, up from 11.1% when using no panel (Figure 5).

### Pathways and Interactions analysis

**Figure 6:**
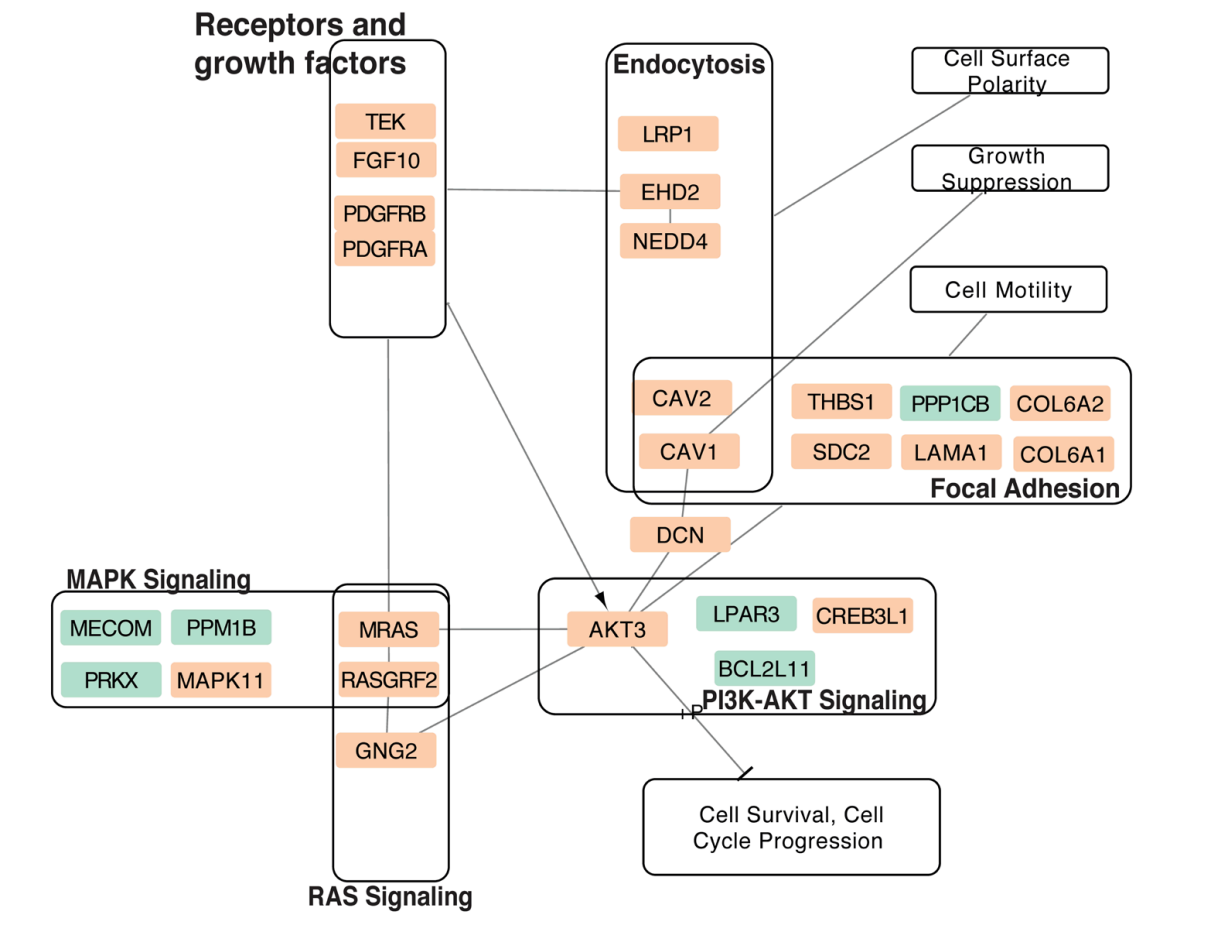
Overlap of enriched pathways from MT vs. NE differential expression Multiple pathways either directly or indirectly regulate AKT3, which is also dysregulated in BT as compared to MT (Table 3). In addition, the receptors and growth factor affect a majority of enriched pathways. Orange nodes represent downregulated transcripts, and the green nodes represent upregulated transcripts. STRING- DB dataset was used to include additional Protein-Protein Interactions (supplementary material).

Pathway enrichment analysis using the 26-genes signature panel (Table 3) identified 10 KEGG annotated human pathways. The results show dysregulation of critical cellular mechanisms and signaling pathways. The dysregulated pathways include endocytosis, focal adhesion, MAPK signaling, PI3K-AKT signaling, and RAS signaling (Table 5). P-values of enrichment analysis are calculated using chi-squared test. The analysis was carried through Partek Software Package and KEGG pathway database. The overlaps of the identified pathways are summarized as a network of dysregulation, with some additional interactions included from STRING database. Figure 6 displays the network and illustrates how each module participates in the enriched pathways and biological functions. Connections of elements are either direct molecular interactions or mediated interactions. Some of the dysregulated genes are shared between multiple pathways. For instance, Caveolins (CAV1, CAV2) are shared between endocytosis and focal adhesion. AKT3, CAV1, CAV2, Platelet Derived Growth Factor Receptor A (PDGFRA), and PDGFRB are shared between four pathways. Those include focal adhesion MAPK, PI3K-AKT, and RAS signaling (Figure 6).

**Table 5:** Pathway Enrichment Analysis of differentially expressed genes comparing NE and MT

Pathway Name	P-value	Percentage of pathway genes dysregulated	Number of dysregulated genes in pathway
Proteoglycans in cancer	4.13E-07	5.38	12
Focal adhesion	1.98E-05	4.93	10
Dopaminergic synapse	9.97E-05	5.47	7
PI3K-Akt signaling pathway	0.00013	3.83	13
Rap1 signaling pathway	0.00034	4.32	9
MAPK signaling pathway	0.00065	3.91	10
Ras signaling pathway	0.00084	4.04	9
Amphetamine addiction	0.0014	6.06	4
Vitamin B6 metabolism	0.0016	16.7	1
Endocytosis	0.0018	4.00	8
Melanoma	0.0024	5.71	4

This analysis demonstrates that AKT3 and PDGFRA appear as the central elements in terms of the position in the network, overlaps between the dysregulated pathways, and the fold change magnitude. The results show mRNA downregulation of PDGFRA in MT. Lower mRNA expression of AKT3 are observable in MT compared to BT and NE (Table 2 and Table 3). AKT3 is involved in 8 of the 10 identified pathways – Dopaminergic synapse, Rap1 signaling, Focal adhesion, MAPK, Melanoma, Proteoglycans in cancer, PI3K-AKT, and Ras signaling (Table 5). PDGFRA is associated with 7 of the pathways (supplementary material). Two of the dysregulated genes in MT compared to BT, AKT3 and BCL11, are also involved in the extracted network.

## DISCUSSION

We conducted a comparative analysis of mRNA expression using laser capture micro-dissected ovarian tissues from NE, BT, and MT. Differential expression analysis identifies dysregulated genes that stratify MT from both NE and BT (Tables 2 and 3). The set of four genes AKT3, PTTG1, CENPF, and MND1 are the most significant, based on fold changes, among the dysregulated genes in MT compared to BT and NE (Figure 1). To verify this observation, we utilized two independent publicly available datasets (GSE14407 and GSE9899) (Figure 2). Cross-examination results show statistically significant evidence of consistent dysregulations of four genes (Figure 2). The distinguishing expression patterns of these genes, between the different pathological states, suggest potential roles as indicators of HGSOC malignancy. Prior studies support the roles of some of these genes in ovarian cancer. PTTG1 is a therapeutic target in ovarian cancer and is associated with functions including DNA repair, angiogenesis, and cell development [22, 23]. CENPF expression has been associated with cell cycle progression, and malignancy through FOXM1 [20, 24]. To our knowledge, AKT3 downregulation and MND1 upregulation have not been previously reported in human HGSOC.

While dysregulations of the set of fours genes are significant between MT vs BT and NE, there are also some detectable dysregulation between BT and NE (Figures 1 and 2B). As the dysregulations indicate the tumor presence, their intensity can determine whether the tumors are malignant or not. Moderate dysregulation of BT as compared to NE is supported by the fact that benign tumors exhibit functional similarities with malignant tumors, including evading growth suppression and resisting cell death [14, 16]. However, malignant tumors are expected to exhibit more extreme dysfunctions as well as other unique characteristics such as metastasis [14]. In our study, enrichment analysis results of the 26 dysregulated signature gene panel (MT vs BT and NE) identified G2M checkpoint and E2F transcription factor cell cycle-related targets, which are related with P53 activities [25, 26]. These results are supported by the reports of HGSOC displaying P53 mutations in more than 70% of cases [1, 27], compared to dramatically lower rates in low malignant potential tumors [28].

The classification results display the utility of the 26-gene signature panel to distinguish between MT and both BT and NE when the signals are combined (Figure 4 and 5, Table 4). PCA combines mRNA signals and represent the microarray samples in a lower dimensional space, which allows for an interpretable feature selection. k-means clustering shows that the 26-genes panel stratify the PCA-projected samples, based on their respective tissue types (Figures 4A and 5A). In a similar procedure, without filtering the input mRNA signals, the classification algorithm loses its stratification power (Table 4). This observation indicates that the comparative analysis of LCM samples generates a focused and meaningful outline of the genetic dysregulations necessary for identification of HGSOC. As HGSOC is known to encompass a diverse genetic profile, the results show how the use of mRNA panel boosts the classification quality and addresses the issue of genetic heterogeneity. This also indicates that the signature mRNA panel can contribute to the design of future collective biomarkers as alternative to single- molecule biomarkers.

Pathway enrichment analysis identifies functional associations of the dysregulated genes, and shows major overlaps among the ten enriched KEGG pathways (Table 5, Figure 6). In particular, AKT3 appears in 8 and PDGFRA appears in 7 of the 10 pathways. PDGFRA is connected to AKT3 activity and regulations in participates in cancer related mechanisms [29]. PDGFRA alteration is associated with ovarian cancer but not frequently mutated [30, 31]. This suggests that the interconnections between AKT3 and PDGFRA are potential for future studies. The extracted network of pathway overlaps shows associations of HGSOC dysregulation with several critical cancer mechanisms, including cell survival, cell motility, and tumor growth. Additional evidence from the literature indicates that endocytosis and focal adhesion components of this network are involved in Epithelial-Mesenchymal Transition (EMT), and cell motility [32-34].

Based on the dysregulation patterns and functional associations, our results indicate that AKT3 is a potential central marker of tumor presence and malignancy. AKT3 is one of the 3 major protein kinase B isoforms, and exhibits high levels of homology to the other members of the AKT family (AKT1 and AKT2), particularly in the highly conserved phosphorylation sites [35]. AKTs are frequently dysregulated in ovarian cancer, and participate in various cancer associated activities, including proliferation, migration, survival, and apoptosis [35-41]. While AKT3 is relatively understudied compared to the other members of AKT family, its downregulation is to some extent contrary to the early literature [39, 42, 43]. Earlier studies reported upregulation of AKT molecules in breast, prostate, and ovarian cancers [35, 39, 42, 43]. However, our results show that AKT3 is downregulated, which is also supported by more recent studies. Phung et al. showed that AKT3 inhibits growth in vascular tumors and displays declined expression [40]. Grottke et al. also showed that AKT3 downregulation results in elevated migration and metastasis in breast cancer [44]. Based on validation in independent public datasets (GSE9899 and GSE14407), we conclude that AKT3 downregulation in HGSOC implies opposing patterns compared to reports of AKT1 and AKT2 [20]. In addition, we identify AKT3 as a potential central marker of tumor malignancy, as its levels in BT are slightly lower than in NE, and significantly further decreased in MT compared to BT (Figure 2). Nakatani et al. reported that AKT3 enzyme activity levels correlate with its upregulated mRNA levels in breast cancer and prostate cancer [39]. This finding, along with our results, suggests that AKT3 mRNA downregulation could reduce its protein phosphorylation activity and potentially can be used to establish a strong HGSOC biomarker. In addition, AKT3 downregulations might contribute to tumor growth, metastasis, and cell migration in HGSOC [40, 44]. We conclude that further study of AKT3 activities in ovarian cancer will lead to new insights of the signaling activities, especially those related to the PI3K-AKT pathway.

The results presented in this article display how the comparative investigation of mRNAs provides signatures for identifying HGSOC relative to both normal epithelia and benign tumors. Coupled with pathway analysis, we determine functional dysregulations associated with these signatures. The activities of the dysregulated genes and their causal relationships need to be further investigated using other molecular aspects such as protein assays, DNA methylation, and phosphorylation. The significant changes in mRNA levels of the 26-gene signature panel indicate their potential as biomarkers for HGSOC, due to their specificity to malignant tissues. We showed how the dysregulated genes could be used as molecular signatures to stratify HGSOC (Figure 2). These molecular signatures can potentially be used to enhance early detection for HGSOC and ultimately contribute to managing mortality rates.

## MATERIALS AND METHODS

Our initial in-house data set consists of 24 cell specific samples processed through LCM, 10 normal epithelial tissues (NE), 8 malignant tumor tissues (MT), and 6 benign tumor tissues (BT) (Table 1). In addition, we utilized two related and independently collected datasets for verification of results in larger datasets, GSE14407 and GSE9899. GSE14407 consists of 12 normal ovarian epithelial tissues and 12 ovarian malignant tumor tissues. GSE9899 includes 18 low malignant potential samples and 246 HGSOC samples. Our in-house data is publicly available through NCBI Gene Expression Omnibus using reference code GSE 29156. Non-tumorous ovarian epithelial tissues, and tumor tissues from benign and malignant serous carcinoma samples were analyzed with GeneChip Affymetrix Human Exon 1.0 ST arrays. Gene expressions were subsequently subjected to multiple statistical procedures for production of accurate and quality results.

### Tissue collection

Ovarian specimens were obtained, under IRB approved guidelines at Carolinas Medical Center (CMC), during surgery for ovarian cancer or other gynecologic conditions. The samples were classified as normal ovary, benign tumors, or malignant tumors. Malignant tissues were from patients with serous carcinoma at stage II or III, grade 3 according to pathological diagnosis assigned by pathologists at CMC using the World Health Organization criteria for ovarian tumors (Table 1). Tissue samples were placed in a standard sized cryomold (Sakura Finetek USA, Inc., Torrance, CA), covered with Optimal Cutting Temperature (OCT) compound (Sakura Finetek USA, Inc., Torrance, CA), frozen and stored at -80°C [19].

### Laser Capture Microdissection (LCM)

OCT embedded samples were serially sectioned into 8μm sections and prepared for LCM using the HistoGene LCM Frozen Section Staining kit (Applied Biosystems, Life Technologies, Co., Carlsbad, CA) according to the manufacturer's protocols. The stained sections were immediately micro-dissected by an Arcturus® PixCell® IIe LCM (Molecular Devices, LLC, Sunnyvale, CA). Normal epithelium and tumor cells were separately collected from appropriate sections. RNA quality was determined using the residual slide materials [19].

### cDNA Synthesis and Amplification

Single primer isothermal amplified (SPIA) complimentary DNA (cDNA) was generated and amplified using the Whole Transcriptome WT-Ovation Pico RNA Amplification System Kit (NuGEN Technologies Inc., San Carlos, CA), and was used for microarray sample preparation.

### Exon Microarray and Sample Hybridization

3 μg of SPIA amplified cDNA was used to do the sense–strand cDNA (ST-cDNA) conversion using the WT- Ovation Exon Module (NuGEN Technologies Inc., San Carlos, CA). 5 μg ST-cDNA was fragmented and labeled with FL-Ovation cDNA Biotin Module V2 kit (NuGEN Technologies Inc., San Carlos, CA) and hybridized using Affymetrix Human Exon 1.0 ST arrays (Affymetrix, Inc., Santa Clara, CA). Microarray hybridization and scanning was performed using GeneChip Hybridization Oven 640, GeneChip Fluidics Station 450, and GeneChip Scanner 3000 7G with Autoloader respectively (Affymetrix, Inc., Santa Clara, CA) [19].

### Statistical Analysis

The samples were analyzed using Partek® Genomics Suite® (Partek Inc., St. Louis, MO). Microarray transcripts were normalized using GCRMA method. Exons were summarized to genes expressions using winsorized mean (below 10.0% and above 90.0%) and Tukey's biweight one-step. Differential expression p-values were calculated using Analysis of Variance (ANOVA). Unannotated genes were removed and transcripts passed as significant if their False Discovery Rate (FDR) criteria was less than 0.05 (FDR<0.05) (Table 2).

Differentially expressed genes between MT and NE were analyzed to determine differentially expressed genes between BT and MT (FDR < 0.05) (Table 3).

### Validation and confirmation of results

We used two publicly available datasets to verify the results. First, we utilized an experimentally similar datasets from Bowen and colleagues which includes 12 normal ovarian epithelial tissues and 12 ovarian tumor tissues each separated using LCM [9]. The samples were obtained through NCBI GEO portal from GSE14407 dataset. Second, we used whole tissue samples by Tothill and colleagues (GSE9899); with samples limited to Low Malignant Potential (LMP) and ovarian serous carcinoma malignant tumors [12]. The samples of GSE9899 that we used included 18 LMBs and 246 malignant tumors. We used hierarchical clustering algorithm using average linkage and Euclidian distance to cluster the samples from GSE14407 and GSE9899. The hierarchical clustering procedure was done using Partek® Genomics Suite®. We used Principal Component Analysis (PCA) to evaluate the utility of dysregulated genes between MT and BT compared to all gene transcripts in distinguishing between the sample types (Figures 4 and 5). For each test dataset, we separately applied PCA to all annotated genes and the panel of dysregulated genes. PCA was calculated based on the covariance matrix of gene expressions and the data points were shifted to the mean of zero. For further evaluation, we used k-means algorithm for unsupervised clustering of samples into two groups after applying PCA. For each dataset, the results of k-means were calculated based on whether PCA was done on the dysregulated genes between MT and BT or not. PCA results were projected on the first two principle components. Cross validation of results were done using R statistical analysis package [45]. Moreover, the statistical significance of the dysregulated gene between MT and BT with fold change magnitude more than 3 were evaluated in the test datasets (Figure 2).

### Enrichment Analysis

Differentially expressed genes in NE vs MT were further analyzed to investigate the underlying functional dysregulations. Overrepresentation analysis of KEGG annotated pathways was performed using Partek® Genomics Suite®. Enrichment score of each pathway was calculated based on Chi-squared test of differentially expressed gene in pathway, relative to the total number of the annotated genes of pathway (Table 5). P-values of Chi-squared test were subjected to multiple hypothesis testing criteria (FDR < 0.05). Additional interactions were included by manual examination of the Protein-Protein Interactions (PPI) network of Homo Sapiens. PPIs were obtained using STRING-DB online database [46]. The interactions were included if they had medium or higher confidence based on data input of STRING-DB criteria [46]. Gene Ontology (GO) enrichments and related annotated enrichments were investigated based on MSigDB database by Broad Institute [47]. We used the same statistical criteria (FDR < 0.05) as the cut off for reporting enriched terms.

## SUPPLEMENTARY MATERIAL TABLES AND FIGURE





## Abbreviations

BT: Benign Tumors
NE: Normal Epithelia
MT: Malignant Tumors
LCM: Laser Capture Microdissection
LMP: Low Malignant Potential
HGSOC: High-Grade Serous Ovarian Cancer
PCA: Principal Component Analysis
