# Supplementary material for: Dysregulation of AKT3 along with a small panel of mRNAs stratifies high-grade serous ovarian cancer from both normal epithelia and benign tumor tissues
Source: Genes Cancer. 2017 Nov;8(11-12):784–98. doi: 10.18632/genesandcancer.164 (PMC5755724; doi:10.18632/genesandcancer.164)
Supplement: Supplementary file 1 [file ganc-08-784-s001.pdf]

Dysregulation of AKT3 along with a small panel of mRNAs stratifies high-grade serous ovarian cancer from both normal epithelia and benign tumor tissues- Yeganeh et al

Supplementary material

S.1 Pathway information

Pathway enrichment analysis using Partek Software detected statistically significant pathways whose members are overrepresented based on differentially expressed genes list (Supplementary table). This section provides the details of the pathways found using the analysis pipeline. The pathway images are based on KEGG databases (Table S.1.1) and Protein-Protein Interaction (PPI) are collected based on STRING-DB (Figure S.1.1)[1, 2]. Vitamin B6 metabolism was excluded due to very small size of pathway. Amphetamine addiction and Melanoma were also excluded from analysis.

Table S.1.1. Statistically significant enriched pathways based on chi squared test. Third column represents number of pathway genes that were identified as differentially expressed.

Pathway Name	P-value	Genes in list	Genes in pathways	Pathway Reference
Proteoglycans in cancer	4.13E-07	12	211	kegg_pathway_45
Focal adhesion	1.98E-05	10	193	kegg_pathway_188
Dopaminergic synapse	9.97E-05	7	121	kegg_pathway_20
PI3K-Akt signaling pathway	0.000125112	13	326	kegg_pathway_262
Rap1 signaling pathway	0.000341931	9	199	kegg_pathway_206
MAPK signaling pathway	0.000650836	10	246	kegg_pathway_261
Ras signaling pathway	0.000838337	9	214	kegg_pathway_265
Amphetamine addiction	0.00143196	4	62	kegg_pathway_114
Vitamin B6 metabolism	0.00164704	1	5	kegg_pathway_76
Endocytosis	0.00184759	8	192	kegg_pathway_232
Melanoma	0.00237519	4	66	kegg_pathway_27

Figure S.1.1. For each enriched KEGG pathway, its associated dysregulated genes (NE vs MT) were examined through STRING-DB in order to additional interactions. A) Endocytosis. B) Focal Adhesion. C) Proteoglycans in cancer. D) MAPK Signaling. E) PI3K-Akt F) Proteoglycans in cancer. F) RAP1 Signaling G) RAS Signaling.

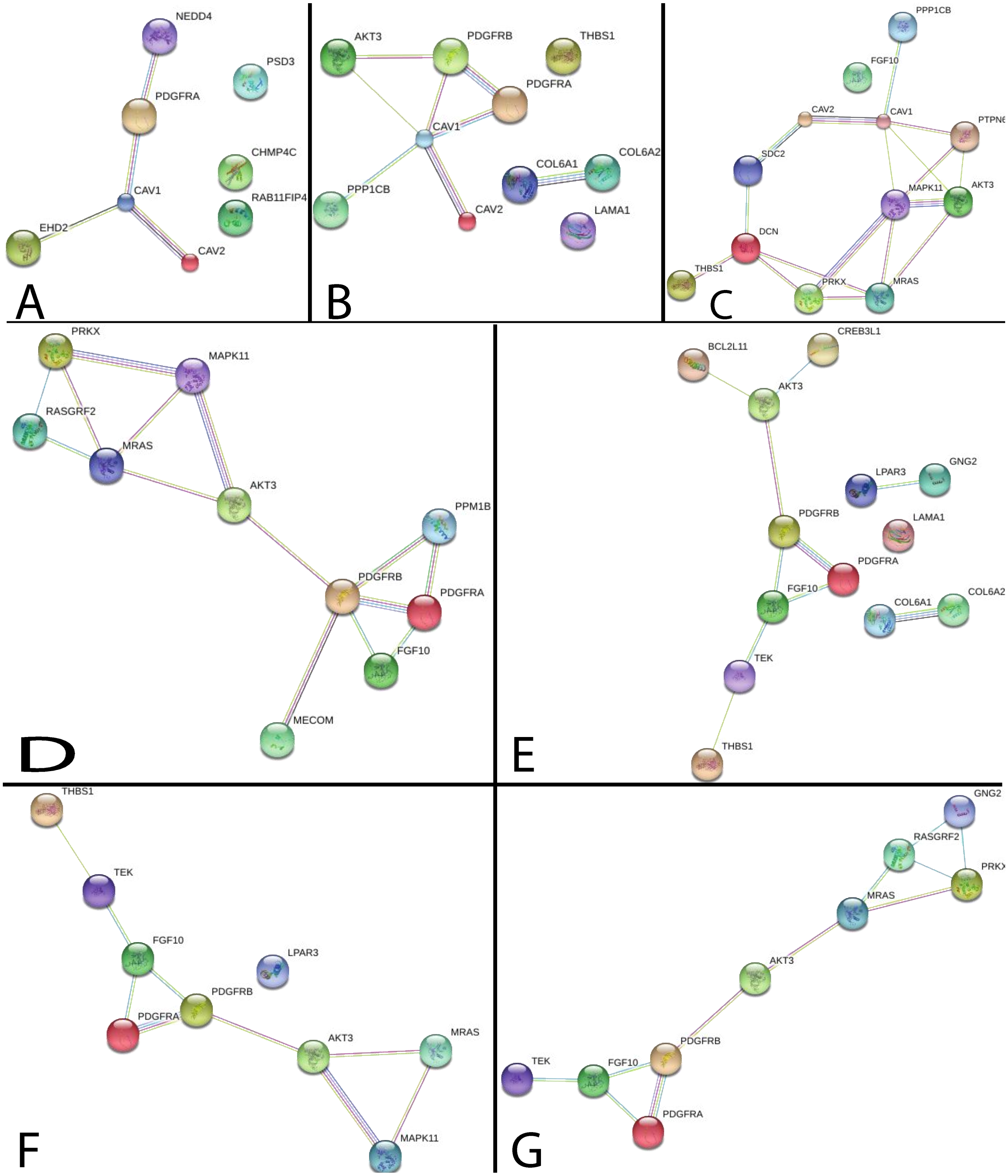

S.2 Cross validation

To validate our results, we used two datasets from Tothill et al. and Bowen et al. [3, 4]. Microarray transcripts were normalized using RMA method. P value for differential expression were calculated using ANOVA. Following tables display p values for differential expression used in figure 2.

Probeset ID	Entrez Gene	Gene Symbol	Gene Title	p-value(Malignant vs. LMP)	Fold-Change(Malignant vs. LMP)
203554_x_at	9232	PTTG1	pituitary tumor-transforming 1	2.83E-19	5.85157
209172_s_at	1063	CENPF	centromere protein F, 350/400kDa	9.90E-11	4.12331
223700_at	84057	MND1	meiotic nuclear divisions 1 homolog (S. cerevisiae)	1.67E-08	3.16569
212607_at		AKT3		0.00117437	-2.08749

Table S.2.1: Differential expression of four genes based on microarray data from Tothill et al dataset [3]. P-values were calculated using ANOVA.

Probeset ID	Entrez Gene	Gene Symbol	Gene Title	p-value(CEPI vs. OSE)	Fold-Change(CEPI vs. OSE)
212607_at	10000	AKT3	v-akt murine thymoma viral oncogene homolog 3	4.11E-06	-5.65894
209172_s_at	1063	CENPF	centromere protein F, 350/400kDa	1.87E-09	5.78085
223700_at	84057	MND1	meiotic nuclear divisions 1 homolog (S. cerevisiae)	0.00750382	1.86361
203554_x_at	9232	PTTG1	pituitary tumor-transforming 1	3.52E-05	4.22071

Table S.2.2: Differential expression of four genes based on microarray data from Bowen et al dataset [4]. P-values were calculated using ANOVA.

References:

1. Kanehisa, M., et al., *KEGG for integration and interpretation of large-scale molecular data sets.* Nucleic acids research, 2011: p. gkr988.
2. Szklarczyk, D., et al., *STRING v10: protein–protein interaction networks, integrated over the tree of life.* Nucleic acids research, 2014: p. gku1003.
3. Tothill, R.W., et al., *Novel molecular subtypes of serous and endometrioid ovarian cancer linked to clinical outcome.* Clinical Cancer Research, 2008. **14**(16): p. 5198-5208.
4. Bowen, N.J., et al., *Gene expression profiling supports the hypothesis that human ovarian surface epithelia are multipotent and capable of serving as ovarian cancer initiating cells.* BMC medical genomics, 2009. **2**(1): p. 1.
